# Water Ingestion in Postural Orthostatic Tachycardia Syndrome: A Feasible Treatment Option?

**DOI:** 10.19102/icrm.2019.100207

**Published:** 2019-02-15

**Keywords:** Autonomic dysfunction, postural orthostatic tachycardia syndrome, syncope

## Dr. Mehdirad remarks

### Mechanisms and symptoms

As noted by Ziffra et al.,^1^ although not completely understood, the pathophysiology of postural orthostatic tachycardia syndrome (POTS) appears to be multifactorial. Autonomic nervous system dysfunction,^2^ increased sympathetic tone,^3^ and physical deconditioning^4,5^ are some of the suggested contributory factors. As such, the recording of a detailed history and the performance of a thorough physical examination including measuring orthostatic parameters are crucially important.

Physiologically, in the setting of a normal resting blood pressure, baroreceptors (stretch receptors in the aortic arch and carotid sinuses) continuously modulate autonomic activity by relaying their signals/impulses to the solitary nucleus in the brainstem as a measure of blood pressure.

An elevation in blood pressure activates the solitary nucleus, which, in turn, inhibits the sympathetic and activates the parasympathetic nervous systems, respectively, resulting in a reduction in blood pressure and heart rate. Inversely, a reduction in blood pressure inhibits the solitary nucleus, leading to the activation of the sympathetic nervous system and the inhibition of the parasympathetic nervous system, causing blood pressure and heart rate to rise.

In light of this, impaired baroreceptor function may play an important role in the pathophysiology of POTS.^6,7^

Clinical manifestations of POTS were well-described by Ziffra et al.^1^ It is noteworthy to mention additionally though that it is not uncommon for POTS patients to also demonstrate manifestations of other disease states such as hypermobility syndrome,^8^ vascular compression syndrome,^9^ and irritable bowel syndrome.^8^ In addition, some patients may present with cholinergic symptoms such as dry mouth, dry eyes, delayed gastric transit, early satiety, nausea, constipation and Holmes–Adie pupil (ie, dilated pupil that constricts slowly in bright light).^10–13^

Hypovolemia or excessive lower-extremity and splanchnic venous pooling results in inadequate venous return, which may possibly explain some of the manifestations of POTS.^14^ These include Raynaud’s phenomenon in dependent extremities, acrocyanosis, food intolerance, postprandial tachycardia, and irritable bowel syndrome manifestations.^15^ In some POTS patients with concomitant conditions causing MAST cell activation, allergic rhinitis/sinusitis and rash may also be present.^16,17^

### Treatment

Ideally, treatment of POTS should be directed toward the goals of (1) patient education, (2) increasing intravascular volume, (3) improving peripheral vasoconstriction, and (4) controlling heart rate to improve symptoms.

Affected patients should be told that the available treatments for POTS may just only improve and not necessarily eradicate their symptoms and informed of the importance of their own active role in improving their symptoms by participation in “lifestyle modification” strategies. Importantly, recumbent exercise activities may improve exercise intolerance and prevent deconditioning.^18^

Low intravascular volume can be clinically diagnosed by the presence of dry mucosa; a narrow upright pulse pressure; and concentrated urine despite adequate water ingestion, which may be suggestive of insensible fluid and electrolyte loss. As such, avoiding overheating, the consumption of oral contraceptives containing drospirenone (a progestin with antimineralocorticoid properties), diuretics, and vasodilators is recommended.

In the common form of POTS, patients who experience a “normal adrenergic response” in the upright position may experience symptom resolution with methods such as increasing intravascular volume with intravascular saline (during acute decompensation), taking in liberal amounts of salt and water, wearing “belly button–high” compression stockings, and sleeping with the head of their bed elevated (to a 30-degree angle). Additionally, fludrocortisone^19^ and midodrine^20,21^ can be helpful in some cases, while some centers have reported good results with octreotide (an octapeptide and somatostatin analog) in patients with postprandial tachycardia; however, this medication is expensive and only available in injection form.^20,22^

In the less common presentation of POTS with “hyperadrenergic response” in the upright position, improper baroreceptor function results in sustained high sympathetic tone that may be due to increased production or decreased clearance of norepinephrine. As such, alpha-agonist therapy (eg, midodrine) may be less desirable and, instead, nonselective β-blocker (propranolol^23^) or ivabradine^24^ (approved by the United States Food and Drug Administration for chronic heart failure treatment only) may be helpful.

Interestingly and intriguingly, droxidopa, a norepinephrine prodrug with approval from the United States Food and Drug Administration for use in neurogenic orthostatic hypotension, has been tried in some POTS patients with good results, although the results are variable and further investigation is required.^25^

In patients with orthostatic hypotension and autonomic dysfunction, sleeping in a seated position at night can reduce salt and water loss (natriuresis).^26^ Furthermore, water ingestion (16 ounces) has been shown to acutely elevate blood pressure by 30 mmHg within five minutes.^27^

The mechanism of pressor response to water is not well-defined but appears to be dependent on the volume ingested and may be the result of gastric distension, which, in turn, causes a reflex increase in arterial pressure, a sympathetically-mediated increase in heart rate, and peripheral vascular resistance (ie, the gastrovascular reflex).

Notably, the digestive system has its own local nervous system referred to the “enteric” or “intrinsic” nervous system. The enteric nervous system, along with the sympathetic and parasympathetic nervous systems, can regulate heart rate and blood pressure. The principal components of the enteric nervous system are the “myenteric plexus” (which mainly controls digestive tract motility) and the “submucosa plexus“ (which mainly regulates gastrointestinal blood flow).

In the present case, Ziffra et al. report a fairly novel strategy of acute water (eight ounces) ingestion for heart rate reduction and symptom amelioration in POTS patients. This appears to be an effective, simple, easy, and safe strategy to implement.

Ali Mehdirad, md (ali.mehdirad@health.slu.edu)^1^

^1^Center for Comprehensive Cardiovascular Care, St. Louis University School of Medicine, St. Louis, MO, USA

Dr. Mehdirad reports that he is a speaker for Lundbeck, Bristol-Myers Squibb, and Pfizer Pharmaceuticals.

References1.ZiffraJBOlshanskyBAcute water ingestion as a treatment for postural orthostatic tachycardia syndromeJ Innov Cardiac Rhythm Manage20191023541354410.19102/icrm.2019.100206PMC7252788324777182.GrubbBPKlingenhebenTPostural orthostatic tachycardia syndrome (POTS): etiology, diagnosis and therapyMed Klin (Munich)2000958442446Article in German. [PubMed]1098506510.1007/s0006300500043.FurlanRJacobGSnellMChronic orthostatic intolerance: a disorder with discordant cardiac and vascular sympathetic controlCirculation1998982021542159[PubMed]981587010.1161/01.cir.98.20.21544.MichaelJPOTS: Deconditioning, pseudodeconditioning, both or neitherLecture presented at: the 18th International Symposium & Second Joint Meeting with EFASOctober 10–13, 2007Vienna, Austria5.JoynerMJMasukiSPOTS versus deconditioning: the same or different?Clin Auton Res2008186300307[CrossRef][PubMed]1870462110.1007/s10286-008-0487-7PMC37702936.BenarrochEEPostural tachycardia syndrome: a heterogeneous and multifactorial disorderMayo Clin Proc2012 Dec871212141225[CrossRef][PubMed]2312267210.1016/j.mayocp.2012.08.013PMC35475467.WellsRSpurrierAJLinzDPostural tachycardia syndrome: current perspectivesVasc Health Risk Manag201714111[CrossRef][PubMed]2934396510.2147/VHRM.S127393PMC57495698.MandelDAskariADMalemudCJKasoAJoint hypermobility syndrome and postural orthostatic tachycardia syndrome (HyPOTS)Biomed Res Clin Prac2017211410.15761/BRCP.10001329.ExelbyGGarrettMVascular compression symdromes in postural orthostatic tachycardia syndrome (POTS)Available at: http://dysautonomia.com.au/vascular-compression-syndromes-in-postural-orthostatic-tachycardia-syndrome-pots/. Accessed August 30, 2018.10.WangLBCulbertsonCJDebAMorgenshternKHuangHHohlerADGastrointestinal dysfunction in postural tachycardia syndromeJ Neurol Sci.20153591–2193196[CrossRef][PubMed]2667111110.1016/j.jns.2015.10.05211.SandroniPOpfer-GehrkingTLMcPheeBRLowPAPostural tachycardia syndrome: clinical features and follow-up studyMayo Clin Proc1999741111061110[CrossRef][PubMed]1056059710.4065/74.11.110612.JohnsonRHMcLellanDLLoveDROrthostatic hypotension and the Holmes-Adie syndrome. A study of two patients with afferent baroreceptor blockJ Neurol Neurosurg Psychiatry1971345562570[CrossRef][PubMed]512238410.1136/jnnp.34.5.562PMC49387013.MehrSEBarbulAShibaoCAGastrointestinal symptoms in postural tachycardia syndrome: a systematic reviewClin Auton Res2018284411421[CrossRef][PubMed]2954945810.1007/s10286-018-0519-xPMC631449014.FreemanRLirofonisVFarquharWBRiskMLimb venous compliance in patients with idiopathic orthostatic intolerance and postural tachycardiaJ Appl Physiol (1985)2002932636644[CrossRef][PubMed]1213387410.1152/japplphysiol.00817.200115.HuangHDebACulbertsonCMorgenshternKDePold HohlerADermatological manifestations of postural tachycardia syndrome are common and diverseJ Clin Neurol20161217578[CrossRef][PubMed]2661089310.3988/jcn.2016.12.1.75PMC471228916.LiHYuXLilesCAutoimmune basis for postural tachycardia syndromeJ Am Heart Assoc201431e000755[CrossRef][PubMed]2457225710.1161/JAHA.113.000755PMC395971717.CheungIVadasPA new disease cluster: mast cell activation syndrome, postural orthostatic tachycardia syndrome, and Ehlers-Danlos syndromeJ Allergy Clin Immunol.20151352 SupplAB6510.1016/j.jaci.2014.12.114618.FuQLevineBDExercise in the postural orthostatic tachycardia syndromeAutonom Neurosci20151888689[CrossRef][PubMed]10.1016/j.autneu.2014.11.008PMC43366032548755119.FreitasJSantosRAzevedoECostaOCarvalhoMde FreitasAFClinical improvement in patients with orthostatic intolerance after treatment with bisoprolol and fludrocortisoneClin Auton Res2000105293299[PubMed]1119848510.1007/BF0228111220.RossAJOconAJMedowMSStewartJMA double-blind placebo-controlled cross-over study of the vascular effects of midodrine in neuropathic compared with hyperadrenergic postural tachycardia syndromeClin Sci (Lond)20141264289296[CrossRef][PubMed]2397822210.1042/CS20130222PMC389607521.HoeldtkeRDBrynerKDHoeldtkeMEHobbsGTreatment of postural tachycardia syndrome: a comparison of octreotide and midodrineClin Auton Res2006166390395[CrossRef][PubMed]1703617710.1007/s10286-006-0373-022.HoeldtkeRDBrynerKDHoeldtkeMEHobbsGTreatment of autonomic neuropathy, postural tachycardia and orthostatic syncope with octreotide LARClin Auton Res2007176334340[CrossRef][PubMed]1776737910.1007/s10286-007-0436-x23.RajSRBlackBKBiaggioniIPropranolol decreases tachycardia and improves symptoms in the postural tachycardia syndrome: less is moreCirculation20091209725734[CrossRef][PubMed]1968735910.1161/CIRCULATIONAHA.108.846501PMC275865024.RuziehMSirianniNAmmariZIvabradine in the treatment of postural tachycardia syndrome (POTS), a single center experiencePacing Clin Electrophysiol2017401112421245[CrossRef][PubMed]2884615110.1111/pace.1318225.RuziehMDasaOPacentaAKarabinBGrubbBDroxidopa in the treatment of postural orthostatic tachycardia syndromeAm J Ther2017242e157e161[CrossRef][PubMed]2756380110.1097/MJT.000000000000046826.BannisterRSeverPGrossMCardiovascular reflexes and biochemical responses in progressive autonomic failureBrain19771002327344[CrossRef]88448710.1093/brain/100.2.32727.RajSRBiaggioniIBlackBKSodium paradoxically reduces the gastropressor response in patients with orthostatic hypotensionHypertension2006482329334[CrossRef][PubMed]1678533210.1161/01.HYP.0000229906.27330.4f

## Dr. Feigofsky examines

POTS is defined as an increase in the heart rate of ≥ 30 bpm, within 10 minutes of standing, in the absence of orthostatic hypotension.^1^ Symptoms associated with severe orthostatic intolerance can include dizziness, presyncope, syncope, palpitations, nausea, and headache. For some patients, these symptoms can become debilitating and severely affect their quality of life. Patients with POTS are generally young women between the ages of 15 years and 25 years. While the definite pathophysiology remains unknown, POTS is sometimes associated with chronic fatigue syndrome,^2^ several autoimmune disorders,^3^ Ehlers–Danlos syndrome type 3,^4–6^ mast cell activation syndrome,^6,7^ and hyperadrenergic states.^7^

Drs. Ziffra and Olshansky^8^ present a case of a 24-year-old nurse with a history of infrequent syncope, palpitations, and tachycardia. A loop recorder was implanted and showed episodes of symptomatic tachycardia. In the clinical setting, tachycardia was reproduced with an upright posture, confirming the diagnosis of POTS. Acute water ingestion performed in the clinic setting demonstrated reproducible suppression of her upright tachycardia based on real-time loop-recorder interrogation.

Acute water ingestion has been described previously as a treatment for autonomic failure.^9^ The presumed mechanism is the activation of the sympathetic nervous system, resulting in increased peripheral vascular resistance, and may involve the vanilloid receptor (TRPV4).^10^ This effect generally occurs within five minutes of water ingestion, peaks at approximately 30 minutes to 40 minutes following ingestion, and can be sustained for an hour or more.

One study^11^ involving healthy subjects showed an improvement in orthostatic tolerance following the ingestion of 16 ounces of room-temperature tap water. Notably, in addition to an improvement in orthostatic tolerance, there was also an attenuation of the increase in heart rate associated with head-up tilt-table testing.^11^ Another study evaluated the effects of acute water ingestion in POTS patients using head-up tilt-table testing, revealing a symptomatic improvement in orthostatic tolerance but no attenuation of the excessive heart rate increase.^12^ Interestingly, in this same study, the use of a clear soup had a worse outcome on orthostatic tolerance in both multiple system atrophy and POTS patients.

POTS is unique to the field of electrophysiology, as the mainstay of therapy involves a nonpharmacologic approach. Invasive strategies are potentially harmful.^1^ Patient participation is imperative to the success of therapy. Exercise in a recumbent or semirecumbent position for 20 minutes to 30 minutes three times a week has been proven to be effective in improving orthostatic tolerance.^13^ The current guidelines consider recommending a daily ingestion of 10 g to 12 g of sodium and 2 L to 3 L of water.^1^

In the present case, there was immediate positive feedback that suggested acute water ingestion improved the patient’s upright heart rate and, presumably, her symptoms. However, a question remains—was this enough positive feedback to encourage repeated water ingestion before standing and continued long-term improvement?

The degree of heart rate attenuation seen in this case has not been described previously. Furthermore, the amount of water used was half of that previously studied (eight ounces versus 16 ounces). It is certainly feasible that the therapy utilized in this case report could be studied among a larger POTS population. Water is universally available, affordable, and easily implemented. Monitoring the acute effects can be done in an outpatient setting, minimizing cost and potentially improving outcomes.

Smartwatches are becoming increasingly popular, especially among younger individuals. As POTS affects primarily younger women, this is an exciting adjunct to patient management in a complex syndrome. Smartwatches use photoplethysmography to access pulse rate. The accuracy of heart rate detection in sinus rhythm has been established, while the accuracy of atrial fibrillation algorithms are a bit more controversial.^14^ In patients without syncope, smartwatches, rather than implanted monitors, would allow patients to see the real-time the effects of a simple intervention, thereby providing enough positive feedback to encourage long-term compliance with nonpharmacologic recommendations. Using smartwatches in the management of POTS is a potential application of this technology not previously entertained. Whether this will be adapted for future research or not, however, remains to be seen.

The care of patients with POTS is often challenging and time-consuming. There is no “one-size-fits-all” approach, and patients must be motivated to participate in their care. Contrary to other disease states in electrophysiology, the use of pharmacotherapy and/or invasive strategies is not the first approach. Until there are larger, randomized clinical trials, it is safe to recommend acute water ingestion prior to assuming an upright posture, as this case demonstrates that such may be quite effective in improving or eliminating symptoms.

Suzy Feigofsky, md (sfeigofsky@iowaheart.com)^1^

^1^Iowa Heart Center, Carroll, IA, USA

Dr. Feigofsky is on the speaker’s bureau for Lundbeck.

References1.SheldonRSGrubbBPOlshanskyB2015 Heart Rhythm Society expert consensus statement on the diagnosis and treatment of postural tachycardia syndrome, inappropriate sinus tachycardia, and vasovagal syncopeHeart Rhythm2015126e41e63[CrossRef][PubMed]2598057610.1016/j.hrthm.2015.03.029PMC52679482.OkamotoLERajSRPeltierANeurohumoral and haemodynamic profile in postural tachycardia and chronic fatigue syndromesClin Sci (Lond)20121224183192[CrossRef][PubMed]2190602910.1042/CS20110200PMC32034113.BlitshteynSAutoimmune markers and autoimmune disorders in patients with postural tachycardia syndrome (POTS)Lupus2015241313641369[CrossRef][PubMed]2603834410.1177/09612033155875664.RomaMMardenCLDe WandeleIPostural tachycardia syndrome and other forms of orthostatic intolerance in Ehlers-Danlos syndromeAuton Neurosci2018 Mar 5piiS1566-0702(17)30298-9[CrossRef][PubMed]10.1016/j.autneu.2018.02.006295196415.ChengJLAuJSGuzmanJCMorilloCAMacDonaldMJCardiovascular profile in postural orthostatic tachycardia syndrome and Ehlers-Danlos syndrome type IIIClin Auton Res2017272113116[CrossRef][PubMed]2800518910.1007/s10286-016-0392-46.Bonamichi-SantosRYoshimi-KanamoriKGiavina-BianchiPAunMVAssociation of postural tachycardia syndrome and Ehlers-Danlos syndrome with mast cell activation disordersImmunol Allergy Clin North Am2018383497504[CrossRef][PubMed]3000746610.1016/j.iac.2018.04.0047.ShibaoCArzubiagaCRobertsLJ2ndHyperadrenergic postural tachycardia syndrome in mast cell activation disordersHypertension2005453385390[CrossRef][PubMed]1571078210.1161/01.HYP.0000158259.68614.408.ZiffraJBOlshanskyBAcute water ingestion as a treatment for postural orthostatic tachycardia syndromeJ Innov Cardiac Rhythm Manage20191023541354410.19102/icrm.2019.100206PMC7252788324777189.YoungTMMathiasCJThe effects of water ingestion on orthostatic hypotension in two groups of chronic autonomic failure: multiple system atrophy and pure autonomic failureJ Neurol Neurosurg Psychiatry2004751217371741[CrossRef][PubMed]1554849310.1136/jnnp.2004.038471PMC173885710.MayMJordanJThe osmopressor response to water drinkingAm J Physiol Regul Integr Comp Physiol20113001R40R46[CrossRef][PubMed]2104807610.1152/ajpregu.00544.201011.LuCDiedrichATungCWater ingestion as prophylaxis against syncopeCirculation20031082126602665[PubMed]1462380710.1161/01.CIR.0000101966.24899.CB12.GraggenWJHessCWHummAMAcute fluid ingestion in the treatment of orthostatic intolerance-important implications for daily practiceEur J Neurol2010171113701376[CrossRef][PubMed]2041229510.1111/j.1468-1331.2010.03030.x13.FuQLevineBDExercise in the postural orthostatic tachycardia syndromeAutonom Neurosci20151888689[CrossRef][PubMed]10.1016/j.autneu.2014.11.008PMC43366032548755114.KoshyASajeevJNerlekarNSmart watches for heart rate assessment in atrial arrhythmiasInt J Cardiol2018266124127[CrossRef][PubMed]2988742810.1016/j.ijcard.2018.02.073

## Ms. Lei, Ms. Sheikh, and Dr. Raj consider

POTS is defined by the presence of frequent and chronic symptoms of orthostatic intolerance that occur and worsen upon upright posture. These symptoms must be accompanied by a sustained heart rate increase of ≥ 30 bpm with standing (or that of ≥ 40 bpm in patients younger than 19 years of age) and that occurs in the absence of hypotension.^1^ It is emphasized that both typical symptoms and hemodynamic criteria must be present for the diagnosis of POTS.

The case study by Ziffra and Olshansky reports on a patient with orthostatic tachycardia upon standing that resolved with the ingestion of eight ounces (240 mL) of water prior to the exercise.^2^ The diagnosis of POTS in this patient is unclear based on the presented heart rate tracing (the authors’ **Figure 1**), despite the noted onset of symptoms upon orthostatic tachycardia. While the resting heart rate was reported to be 75 bpm, this was not apparent in the provided heart rate tracing, which appears to be sustained just below 100 bpm. The heart rate appears to immediately increase to nearly 150 bpm upon standing, but subsequently fall to 120 bpm. An initial heart rate increase is expected upon postural change, but the excessive rapid transient increase presented in the case study could represent a tachycardia variant of “initial orthostatic hypotension” rather than POTS.^3^ The blood pressure response to standing is not shown. Additionally, the patient’s heart rate response after water ingestion is not presented, making it difficult to properly compare the effects of water therapy. As a result, one cannot be certain that the patient meets the diagnostic criteria for POTS or that the water response was clinically relevant in this case.

The effective use of water ingestion as an acute treatment for orthostatic intolerance has been studied in patients with primary autonomic failure and healthy subjects. Given that drinking 500 mL of water does not change plasma volume by more than about 1%,^4^ it is unlikely that orthostatic improvement subsequent to water ingestion is driven by volume augmentation, regardless of one’s health state. Jordan et al. showed that drinking 480 mL of water improved orthostatic tolerance in patients with primary autonomic failure by significantly increasing sympathetic activity.^4^ This was associated with a rigorous pressor response and a concomitant decrease in heart rate upon upright posture, with maximal effects occurring at 30 minutes to 35 minutes after water ingestion.^4^ A similar hemodynamic effect was observed in older healthy subjects aged 57 years ± two years, but not in younger healthy subjects (aged 25 years ± one year).^4^ It was found that any pressor response elicited by water ingestion could be abolished with autonomic ganglionic blockade, indicating the importance of sympathetic activation in the observed response and its contribution to improved orthostatic tolerance.^4,5^

Schroeder et al. observed a blunted heart rate response upon standing in healthy subjects at 15 minutes after the ingestion of 500 mL of water, relative to those receiving only 50 mL of water.^6^ With particular regards to the patient presented in this case study, Shannon et al. reported a moderate drop in standing heart rate with no appreciable change in blood pressure in POTS patients at 35 minutes after the ingestion of 480 mL of water, relative to those without water treatment.^7^

Water-induced sympathetic activation is suspected to be mediated by the hypoosmolality of the ingested water. Given that the oral ingestion of the same volume of saline does not elicit a pressor response, osmolality is likely the stimulus of sympathetic activity rather than water temperature or gastric distention.^5,8^ Acute water ingestion results in significant reductions in osmolality in the portal circulation that are relayed by osmosensitive afferent neurons in this area. Physiological shifts in portal osmolality are detected through activation of the transient receptor potential vanilloid cation channel 4 (TRPV4), which is particularly sensitive to osmotic perturbations.^8^ Deletion of TRPV4 has been discovered to abolish the water-induced pressor response despite unchanged levels of osmolality.^8^ It has been suggested that spinal afferents responsible for detecting hepatic portal modulations relay to dorsal root ganglion neurons that then release neuropeptides to alter sympathetic output.^8,9^ However, the limited evidence regarding the effectiveness of water ingestion in POTS patients elucidates the need for further investigation to evaluate its usefulness as an acute therapy and to determine whether or not the portal osmopressor mechanism that governs the water response is physiologically relevant in POTS.

Lasting improvements in POTS symptoms rely on therapy regimens founded upon lifestyle changes that begin with the maintenance of high fluid and dietary salt intake, with the primary goal of augmenting plasma volume. A high-sodium diet has been shown to expand both total blood volume and plasma volume, concurrently reducing orthostatic tachycardia and upright plasma norepinephrine.^10^ It is also important to avoid the concomitant use of diuretics and other pharmacological agents that may reduce effective blood volume and preload, which may exacerbate tachycardia and other associated symptoms.^11^

Lucy Y. Lei,^1^ Nasia Sheikh, bsc,^1^ and Satish R. Raj, md, msci (satish.raj@ucalgary.ca)^1,2^

^1^Department of Cardiac Sciences, Libin Cardiovascular Institute of Alberta, University of Calgary, Calgary, AB, Canada

^2^Autonomic Dysfunction Center, Division of Clinical Pharmacology, Department of Medicine, Vanderbilt University School of Medicine, Nashville, TN, USA

Dr. Raj reports research grants from the Canadian Institutes of Health Research (about POTS) and the Cardiac Arrhythmia Network of Canada (related to syncope). The other authors report no conflicts of interest for the published content.

References1.SheldonRSGrubbBP2ndOlshanskyB2015 Heart Rhythm Society expert consensus statement on the diagnosis and treatment of postural tachycardia syndrome, inappropriate sinus tachycardia, and vasovagal syncopeHeart Rhythm2015126e41e63[CrossRef][PubMed]2598057610.1016/j.hrthm.2015.03.029PMC52679482.ZiffraJBOlshanskyBAcute water ingestion as a treatment for postural orthostatic tachycardia syndromeJ Innov Cardiac Rhythm Manage20191023541354410.19102/icrm.2019.100206PMC7252788324777183.StewartJMClarkeD“He’s dizzy when he stands up”: an introduction to initial orthostatic hypotensionJ Pediatr20111583499504[CrossRef][PubMed]2097014810.1016/j.jpeds.2010.09.004PMC30294664.JordanJShannonJRBlackBKThe pressor response to water drinking in humans: a sympathetic reflex?Circulation20001015504509[PubMed]1066274710.1161/01.cir.101.5.5045.MayMJordanJThe osmopressor response to water drinkingAm J Physiol Regul Integr Comp Physiol20113001R40R46[CrossRef][PubMed]2104807610.1152/ajpregu.00544.20106.SchroederCBushVENorcliffeLJWater drinking acutely improves orthostatic tolerance in healthy subjectsCirculation20021062228062811[PubMed]1245100710.1161/01.cir.0000038921.64575.d07.ShannonJRDiedrichABiaggioniIWater drinking as a treatment for orthostatic syndromesAm J Med20021125355360[CrossRef][PubMed]1190410910.1016/s0002-9343(02)01025-28.McHughJKellerNRAppalsamyMPortal osmopressor mechanism linked to transient receptor potential vanilloid 4 and blood pressure controlHypertension201055614381443[CrossRef][PubMed]2038596510.1161/HYPERTENSIONAHA.110.151860PMC29653369.LechnerSGMarkworthSPooleKThe molecular and cellular identity of peripheral osmoreceptorsNeuron2011692332344[CrossRef][PubMed]2126247010.1016/j.neuron.2010.12.02810.CeledonioJEGarlandEMNwazueVCEffects of high sdoium intake on blood volume and catecholamines in patients with postural tachycardia syndrome and healthy femalesClin Auton Res20142421111.GarlandEMCeledonioJERajSRPostural tachycardia syndrome: beyond orthostatic intoleranceCurr Neurol Neurosci Rep201515960[CrossRef][PubMed]2619888910.1007/s11910-015-0583-8PMC4664448

## Dr. Kanjwal discusses

POTS can often be challenging and frustrating for the affected patients as well as their treating physicians. Unfortunately, no guidelines-directed therapy for patients with POTS has been established to date. Instead, at this time, all we have is a consensus among experts on various therapies for POTS. In the current issue of the journal, Drs. Ziffra and Olshansky present an interesting case report on the use of the acute oral ingestion of water to ameliorate symptoms of POTS.^1^ The authors have elegantly demonstrated the heart rate and blood pressure responses following the ingestion of eight ounces of water. It is very interesting to note that, in the case, the orthostatic tachycardia along with the symptoms improved following the ingestion of water.

Of note, there is a paucity of literature available on the efficacy of various medical therapies and the available therapies are not always well-tolerated by patients with POTS. The effects of this therapy if reproduced in a large randomized study could be groundbreaking for POTS, as it is economically cheap, readily available, and without any side effects. Acute water ingestion has been previously demonstrated to enhance vagal tone and decrease heart rates in normal people.^2–4^ It is important to note that, although the authors demonstrate acute tachycardia and symptom improvement in the patient discussed, we do not know whether these effects were reproduced on long-term basis. Some authors have also reported that cold and isotonic water elicits more vagotonic effects as compared with saline water.^5^ When administering water therapy, physicians can emphasize that it be cold and without any added salt. The other important thing would be the frequency of this therapy. I recently saw a patient in my office and was able reproduce the results of this case study following the administration of 250 mL of water. I subsequently asked the patient to drink 250 mL of water ever hour for 10 hours. We need to see whether the acute beneficial effects of water ingestion will persist over a prolonged period throughout the day. This will also allow us to evaluate whether acute hemodynamic effects translate into clinical improvement in POTS patients. I would like to congratulate the authors for this innovative case report that could potentially benefit many POTS patients.

Khalil Kanjwal, md, fhrs, facc, ceps, ccds^1^

^1^McLaren Cardiovascular Group, Michigan State University, Lansing, MI, USA

The author reports no conflicts of interest for the published content.

References1.ZiffraJBOlshanskyBAcute water ingestion as a treatment for postural orthostatic tachycardia syndromeJ Innov Cardiac Rhythm Manage20191023541354410.19102/icrm.2019.100101PMC7252788324777182.CallegaroCCMoraesRSNegrãoCEAcute water ingestion increases arterial blood pressure in hypertensive and normotensive subjectsJ Hum Hypertens2007217564570[CrossRef][PubMed]1734490810.1038/sj.jhh.10021883.RoutledgeHCChowdharySCooteJHTownendJNCardiac vagal response to water ingestion in normal human subjectsClin Sci (Lond)20021032157162[CrossRef][PubMed]1214910710.1042/cs10301574.ScottEMGreenwoodJPGilbeySGStokerJBMaryDAWater ingestion increases sympathetic vasoconstrictor discharge in normal human subjectsClin Sci (Lond)20011003335342[CrossRef][PubMed]112221215. BrownCMBarberiniLDullooAGMontaniJPCardiovascular responses to water drinking: does osmolality play a role?Am J Physiol Regul Integr Comp Physiol20052896R1687R1692[CrossRef][PubMed]1603712710.1152/ajpregu.00205.2005

## Dr. Cannom assesses

The case report by Ziffra and Olshansky^1^ describes a 24-year-old patient with POTS whose tachycardia with standing (from 75 bpm to 150 bpm) was eliminated by the ingestion of eight ounces of water. This observation was reproducible, though the duration of the effect was not stated.

There is a surprisingly robust amount of literature in existence that describes the benefits of water ingestion in patients with a variety of autonomic diseases including orthostatic hypotension due to autonomic failure^2,3^ and POTS.^2,4^ However, these investigations were performed by basic scientists and neurologists and have not yet had a significant impact on the clinical care of these patients.

Still, all of these investigators agree that the ingestion of water in a patient with orthostatic hypotension triggers a pressor response that has an onset within five minutes after water consumption with a systolic blood pressure increase of 33 mmHg.^3^ The pressure increase reaches its maximum level at 30 minutes to 40 minutes and is maintained for one hour.^3^ In POTS patients given 480 cc of water before a head-up tilt test, there was little change in their blood pressure with water ingestion, but there was a decrease of 15 bpm in heart rate response after three minutes of standing as compared with in the control group.^2^ There is a consensus that this reflex in both groups of patients is mediated by sympathetic nerves releasing norepinephrine and does not reflect volume expansion. There is also agreement among investigators that the sympathetic reflex effect that raises blood pressure or lowers pulse rate is short-lived.

The case presented by Ziffra and Olshansky^1^ is similar to the small number of existing cases in the literature, although the effects of water ingestion in their patient—which reduced the pulse rate from 150 bpm to 75 bpm—is more marked than in the typical patient in the literature.

The use of water ingestion to control the pulse rate in POTS patients would perhaps require 3,600 cc of water per day, assuming 240 cc would be ingested each hour while awake. There is as of yet no data available regarding what number of POTS patients have this sympathetic reflex with water, what its duration is in a large group of patients, and how well this approach would be tolerated. Many of us care for POTS patients who drink this much water during an average day, but this alone does not usually obviate the need for further treatment.

There have been advances in the care of POTS patients that are generally applicable. In the uncomplicated POTS patient who is symptomatic due to orthostatic tachycardia, the traditional approach has been the use of a β-blocker in association with hydration, salt, and compression stockings. I have found that this approach works in a minority of patients, and the next step is generally to prescribe a β-blocker to control the tachycardia in POTS. However, this therapy is often ineffective or not well-tolerated.

We next make every effort to use ivabradine to treat the tachycardia. This therapy generally results in a better response, is sensitive to increases in dosing, and is well-tolerated long-term. However, a major problem facing POTS patients is the approval of ivabradine by insurance companies, for, without insurance, this is a very expensive treatment. There was no comment about the use of ivabradine in POTS patients in the recently published 2015 Heart Rhythm Society Expert Consensus Statement on the Diagnosis and Treatment of POTS.^5^

Patients with more complicated forms of POTS—which includes those with Ehlers–Danlos syndrome, mast cell activation disease, neuromuscular disease, and various autoimmune diseases—need a complicated pharmacologic approach, and heart rate control is often a minor part of their treatment. However, improving the issues of blood pressure support and heart rate treatment are often ignored, and their treatment can be beneficial in these complicated patients.

David S. Cannom, md^1–3^

^1^Good Samaritan Hospital, Los Angeles, CA, USA

^2^Cedars-Sinai Heart Institute, Los Angeles, CA, USA

^3^David Geffen School of Medicine, University of California, Los Angeles, Los Angeles, CA, USA

The author reports no conflicts of interest for the published content.

References1.ZiffraJBOlshanskyBAcute water ingestion as a treatment for postural orthostatic tachycardia syndromeJ Innov Cardiac Rhythm Manage20191023541354410.19102/icrm.2019.100101PMC7252788324777182.ShannonJRDiedrichABiaggioniIWater drinking as a treatment for orthostatic syndromesAm J Med20021125355360[CrossRef][PubMed]1190410910.1016/s0002-9343(02)01025-23.MayMJordanJThe osmopressor response to water drinkingAm J Physiol Regul Integr Comp Physiol20113001R40R46[CrossRef][PubMed]2104807610.1152/ajpregu.00544.20104.Z’GraggenWJHessCWHummAMAcute fluid ingestion in the treatment of orthostatic intolerance—important implications for daily practiceEur J Neurol2010171113701376[CrossRef][PubMed]2041229510.1111/j.1468-1331.2010.03030.x5.SheldonRSGrubbBPOlshanskyB2015 Heart Rhythm Society expert consensus statement on the diagnosis and treatment of postural tachycardia syndrome, inappropriate sinus tachycardia, and vasovagal syncopeHeart Rhythm2015126e41e63[CrossRef][PubMed]2598057610.1016/j.hrthm.2015.03.029PMC5267948

## References

[1] Ziffra JB, Olshansky B (2019). Acute water ingestion as a treatment for postural orthostatic tachycardia syndrome. J Innov Cardiac Rhythm Manage.

[2] Grubb BP, Klingenheben T (2000). Postural orthostatic tachycardia syndrome (POTS): etiology, diagnosis and therapy. Med Klin (Munich).

[3] Furlan R, Jacob G, Snell M (1998). Chronic orthostatic intolerance: a disorder with discordant cardiac and vascular sympathetic control. Circulation.

[4] Michael J (October 10–13, 2007). POTS: Deconditioning, pseudodeconditioning, both or neither. Lecture presented at: the 18th International Symposium & Second Joint Meeting with EFAS.

[5] Joyner MJ, Masuki S (2008). POTS versus deconditioning: the same or different?. Clin Auton Res.

[6] Benarroch EE (2012 Dec). Postural tachycardia syndrome: a heterogeneous and multifactorial disorder. Mayo Clin Proc.

[7] Wells R, Spurrier AJ, Linz D (2017). Postural tachycardia syndrome: current perspectives. Vasc Health Risk Manag.

[8] Mandel D, Askari AD, Malemud CJ, Kaso A (2017). Joint hypermobility syndrome and postural orthostatic tachycardia syndrome (HyPOTS). Biomed Res Clin Prac.

[9] Exelby G, Garrett M Vascular compression symdromes in postural orthostatic tachycardia syndrome (POTS). http://dysautonomia.com.au/vascular-compression-syndromes-in-postural-orthostatic-tachycardia-syndrome-pots/.

[10] Wang LB, Culbertson CJ, Deb A, Morgenshtern K, Huang H, Hohler AD (2015). Gastrointestinal dysfunction in postural tachycardia syndrome. J Neurol Sci..

[11] Sandroni P, Opfer-Gehrking TL, McPhee BR, Low PA (1999). Postural tachycardia syndrome: clinical features and follow-up study. Mayo Clin Proc.

[12] Johnson RH, McLellan DL, Love DR (1971). Orthostatic hypotension and the Holmes-Adie syndrome. A study of two patients with afferent baroreceptor block. J Neurol Neurosurg Psychiatry.

[13] Mehr SE, Barbul A, Shibao CA (2018). Gastrointestinal symptoms in postural tachycardia syndrome: a systematic review. Clin Auton Res.

[14] Freeman R, Lirofonis V, Farquhar WB, Risk M (2002). Limb venous compliance in patients with idiopathic orthostatic intolerance and postural tachycardia. J Appl Physiol (1985).

[15] Huang H, Deb A, Culbertson C, Morgenshtern K, DePold Hohler A (2016). Dermatological manifestations of postural tachycardia syndrome are common and diverse. J Clin Neurol.

[16] Li H, Yu X, Liles C (2014). Autoimmune basis for postural tachycardia syndrome. J Am Heart Assoc.

[17] Cheung I, Vadas P (2015). A new disease cluster: mast cell activation syndrome, postural orthostatic tachycardia syndrome, and Ehlers-Danlos syndrome. J Allergy Clin Immunol..

[18] Fu Q, Levine BD (2015). Exercise in the postural orthostatic tachycardia syndrome. Autonom Neurosci.

[19] Freitas J, Santos R, Azevedo E, Costa O, Carvalho M, de Freitas AF (2000). Clinical improvement in patients with orthostatic intolerance after treatment with bisoprolol and fludrocortisone. Clin Auton Res.

[20] Ross AJ, Ocon AJ, Medow MS, Stewart JM (2014). A double-blind placebo-controlled cross-over study of the vascular effects of midodrine in neuropathic compared with hyperadrenergic postural tachycardia syndrome. Clin Sci (Lond).

[21] Hoeldtke RD, Bryner KD, Hoeldtke ME, Hobbs G (2006). Treatment of postural tachycardia syndrome: a comparison of octreotide and midodrine. Clin Auton Res.

[22] Hoeldtke RD, Bryner KD, Hoeldtke ME, Hobbs G (2007). Treatment of autonomic neuropathy, postural tachycardia and orthostatic syncope with octreotide LAR. Clin Auton Res.

[23] Raj SR, Black BK, Biaggioni I (2009). Propranolol decreases tachycardia and improves symptoms in the postural tachycardia syndrome: less is more. Circulation.

[24] Ruzieh M, Sirianni N, Ammari Z (2017). Ivabradine in the treatment of postural tachycardia syndrome (POTS), a single center experience. Pacing Clin Electrophysiol.

[25] Ruzieh M, Dasa O, Pacenta A, Karabin B, Grubb B (2017). Droxidopa in the treatment of postural orthostatic tachycardia syndrome. Am J Ther.

[26] Bannister R, Sever P, Gross M (1977). Cardiovascular reflexes and biochemical responses in progressive autonomic failure. Brain.

[27] Raj SR, Biaggioni I, Black BK (2006). Sodium paradoxically reduces the gastropressor response in patients with orthostatic hypotension. Hypertension.

